# Exposure to family violence from childhood to adulthood

**DOI:** 10.1186/s12889-020-09709-y

**Published:** 2020-11-09

**Authors:** Margot Shields, Lil Tonmyr, Wendy E. Hovdestad, Andrea Gonzalez, Harriet MacMillan

**Affiliations:** 1 Public Health Agency of Canada, 785 Carling Ave. 7th floor, Ottawa, ON K1A 0K9 Canada; 2 Department of Psychiatry & Behavioural Neurosciences, McMaster University, 1280 Main Street West - MIP 201A, Hamilton, ON L8S 4K1 Canada; 3 Department of Psychiatry & Behavioural Neurosciences, and of Pediatrics, McMaster University, 1280 Main Street West - MIP 201A, Hamilton, ON L8S 4K1 Canada

**Keywords:** Intimate partner violence, Physical abuse, Sexual abuse, Childhood exposure to intimate partner violence, Domestic violence

## Abstract

**Background:**

Both childhood maltreatment (CM) and intimate partner violence (IPV) are public health problems that have been related to a wide range of adverse health consequences. However, studies examining associations between specific types of CM and experiencing IPV in adulthood have yielded conflicting results.

**Methods:**

Using data from 10,608 men and 11,458 women aged 18 or older from Canada’s 2014 General Social Survey, we examined associations between three types of CM—childhood physical abuse (CPA), childhood sexual abuse (CSA), and childhood exposure to IPV —and subsequent intimate partner violence (IPV) in adulthood (physical, sexual or emotional).

**Results:**

When potential confounders were controlled, CPA, CSA and childhood exposure to IPV were associated with IPV in adulthood for both sexes (odds ratios, 1.7, 1.8 and 2.0 for men, and 2.2, 2.0 and 2.1 for women). When severity and frequency of CM were examined, a dose-response relationship between all three types of CM and IPV in adulthood was observed among women (meaning that as the severity/frequency of CM increased, the likelihood of reporting IPV also increased); among men, a dose-response relationship was observed only for CPA.

**Conclusions:**

The association between CM and IPV in adulthood is particularly concerning because experiencing multiple forms of trauma has cumulative effects. Lifespan studies have shown that individuals who experience multiple incidents of abuse exhibit the highest levels of impairment. This underscores the importance of programs to eradicate both CM and IPV. This underscores the importance of programs to eradicate both CM and IPV. Future research should focus on assessing interventions designed to promote healthy relationships and the provision of emotional support and coping mechanisms to children and families in abusive situations.

## Background

The World Health Organization defines intimate partner violence (IPV) as any “behaviour by an intimate partner that causes physical, sexual or psychological harm, including acts of physical aggression, sexual coercion, psychological abuse and controlling behaviours. This definition covers violence by both current and former spouses and other intimate partners” [1]. IPV victimization is associated with short- and long-term health consequences including injury and other physical health conditions, mental health symptoms and disorders, and death [2]. Those experiencing IPV use proportionately more health care services (primary care, emergency, and hospital), even when confounding factors such as socioeconomic status are taken into account [2].

The causes of IPV are complex. The ecological model contends the multiple risk factors are involved including individual, relationship, community, and societal factors [2–4]. An extensive body of literature has examined childhood maltreatment (CM) as a risk factor for being victimized by IPV in adulthood, with the majority of studies focusing on associations with childhood sexual abuse (CSA) [5–18] and childhood physical abuse (CPA) [5–7, 9, 10, 12–22]. Studies of associations with other types of CM, such as childhood exposure to IPV, emotional abuse, and neglect, are less common [5, 6, 13–20, 22–25]. Many of these studies have samples comprised exclusively of women [5, 9, 10, 13, 14, 17, 19, 20].

However, findings tend to be inconsistent. Some studies have found that CM increases the likelihood of IPV in adulthood; others have reported null associations between specific types of CM and IPV [5–7, 12–14, 19, 20, 22, 24, 26–29]. Two meta-analyses [30, 31] of associations between specific types of CM (CPA, CSA, childhood exposure to IPV, neglect, and emotional abuse) and IPV in adulthood concluded that associations were weak, but statistically significant. One of these studies [30] focused on associations between CM and IPV among men and noted a need for research on different kinds of CM and the co-occurrence of CM in relation to IPV victimization and perpetration among men.

Evidence is also mixed about whether greater severity of CM heightens the risk of IPV in adulthood [5, 9, 12, 15, 18–20, 28, 32]. It has been argued that simply being exposed to CM, regardless of severity, increases the risk for future abuse [33]. Some studies have suggested that the number of *types* of maltreatment that a child experiences is more important in predicting IPV in adulthood than is the severity of a specific form of maltreatment [15, 34].

A Canadian study using data from the population-based 1999 General Social Survey (GSS) found an association between CSA and IPV in adulthood for both sexes, although the relationship was weaker for men [11]. The 1999 GSS identified CSA and CPA with questions about life-time history of sexual and physical assault and age of onset; respondents reporting assault before age 18 were classified as having experienced CSA/CPA [35]. For the 2014 GSS, the questions were broadened to include items to measure CSA, CPA, and childhood exposure to IPV [36]. An analysis of 2014 GSS data observed an association between CSA/CPA and severe IPV in adulthood (being beaten, choked, threatened with a gun or a knife, or forced or manipulated into unwanted sexual activity) [37]. However, this study did not examine if increases in severity of CM augmented the likelihood of adult IPV victimization. Childhood exposure to IPV was not considered nor were sex differences in associations between CM and IPV victimization in adulthood.

This article uses data from the 2014 GSS to meet three objectives:

1. to examine associations between three types of CM (CPA, CSA, and childhood exposure to IPV) and being victimized by three types of IPV in adulthood (physical, sexual and emotional);
2. to investigate whether greater severity and frequency of specific forms of CM and the co-occurrence of different types of CM increase the risk of IPV in adulthood; and
3. to determine if associations between CM and IPV in adulthood differ by sex.

Examining these questions will address important gaps in the literature; associations between CM and IPV have rarely been examined among men and more studies are needed to clarify if the co-occurrence and severity of particular types of CM increases the likelihood of IPV.

In this article we use the terms “experienced” IPV or “reported IPV” to refer to “being victimized” by IPV. The article does not address IPV perpetration (see Limitations).

## Methods

### Data source

Data are from Statistics Canada’s 2014 General Social Survey: Victimization. The GSS target population was household residents aged 15 or older living in the 10 provinces and 3 territories. Two samples were selected (one for the provinces; one for the territories) using complex multistage sampling designs that utilized a sampling frame derived from the census and various administrative sources, which combined landline and cellular telephone numbers. More information about the sample design is available in the GSS Microdata User Guide [38]. The response rate was 52.9% for the provinces (33,127 respondents) and 58.7% for the territories (2040 respondents). The two samples were pooled to produce estimates for all Canadians.

The study population for the present analysis was respondents aged 18 or older currently living with a spouse/partner or who had contact with an ex-spouse/ex-partner in the past 5 years (10,608 men and 11,458 women). Respondents aged 15 to 17 years were excluded since our objective was to examine CM in relation to IPV in adulthood.

### Measures

#### Child maltreatment

**Fig. 1:**
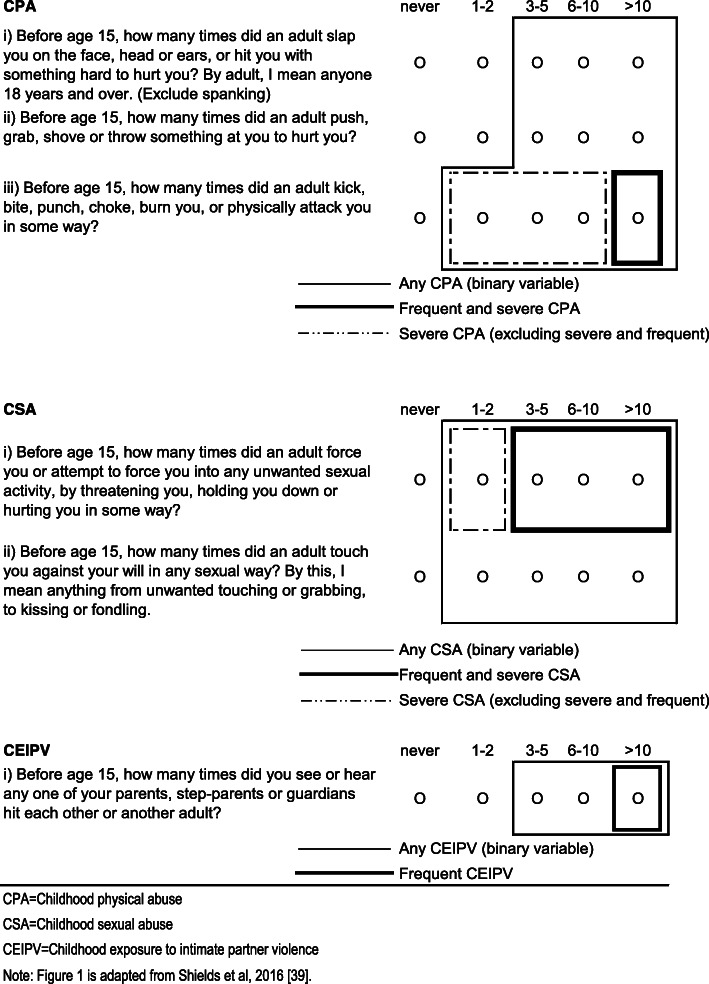
Childhood maltreatment items and definitions. CPA = Childhood physical abuse. CSA = Childhood sexual abuse. CEIPV=Childhood exposure to intimate partner violence. Note: Fig. 1 is adapted from Shields et al., [39]

CPA, CSA, and childhood exposure to IPV were assessed retrospectively by asking respondents about “events that may have happened before you were 15” using the items in Fig. 1 (adapted from Shields et al., [39]).

The items for CPA and childhood exposure to IPV are from the *Childhood Experiences of Violence Questionnaire* (CEVQ) [40]. For each type of abuse, binary variables (yes/no) were created following CEVQ guidelines. As well, variables were derived to indicate the severity and frequency of abuse (Fig. 1). We also examined the co-occurrence of the three types of CM maltreatment as well as each type of CM occurring in isolation. We created a variable to indicate:

- no CM,
- CPA only (no CSA/childhood exposure to IPV),
- CSA only (no CPA/childhood exposure to IPV),
- childhood exposure to IPV only (no CPA/CSA),
- 2 or more types of CM.

We attempted to consider two co-occurrence categories separately (i.e., 2 types and 3 types) but there was insufficient sample size to consider 3 types.

For CPA and CSA, all incidents of abuse were included regardless of the relationship of the perpetrator to the child (e.g., parent, stepparent, other family member, teacher, stranger etc.).

#### Intimate partner violence in adulthood

To identify physical, sexual and emotional IPV in adulthood, questions were asked about the respondent’s current spouse/partner, and then, about the respondent’s ex-spouse/ex-partner (if applicable and if they had contact in the past 5 years). Respondents in same-sex relationships are included in the study. IPV that occurred in dating relationships was excluded. The IPV items are based on the *Conflict Tactics Scales* [41, 42]. Questions about physical and sexual IPV pertained to experiences that occurred over the past 5 years; no time frame was specified for emotional IPV. Table 1 displays the items used to measure IPV.

**Table 1:** Intimate partner violence (IPV) items and definitions

**Physical IPV**: reporting at least one of the following 9 experiences	
During the past 5 years, has your spouse/partner or ex-spouse/ex-partner:	
1 threatened to hit you with his/her fist or anything else that could have hurt you?	
2 thrown anything at you that could have hurt you?	
3 pushed, grabbed or shoved you in a way that could have hurt you?	
4 slapped you?	
5 kicked you, bit you, or hit you with his/her fist?	
6 hit you with something that could have hurt you?	
7 beaten you?	
8 choked you?	
9 used or threatened to use a gun or knife on you?	
**Sexual IPV**: reporting at least one of the following 2 experiences	
During the past 5 years, has your spouse/partner or ex-spouse/ex-partner:	
1 forced you into any unwanted sexual activity, by threatening you, holding you down, or hurting you in some way?	
2 subjected you to a sexual activity to which you were not able to consent. By this I mean were you drugged, intoxicated, manipulated or forced in other ways than physically?	
**Emotional IPV**: reporting at least one of the following 7 experiences	
Your spouse/partner or ex-spouse/ex-partner:	
1 tries to limit your contact with family or friends;	
2 puts you down or calls you names to make you feel bad;	
3 is jealous and doesn’t want you to talk to other men or women;	
4 harms, or threatens to harm, someone close to you;	
5 harms or threatens to harm your pet(s);	
6 demands to know who you are with and where you are at all times;	
7 damages or destroys your possessions or property.	

#### Covariates

Selection of potential confounders to use in the multivariate regression models was based on a review of the literature examining socio-demographic risk factors for CM and IPV [23, 43–47] as well as socio-demographic controls used in other studies investigating CM in relation to IPV in adulthood [5–10, 13–15]. The control variables used in this study include: current age (18–24, 25–39, 40–54, 55–69, 70 or older), born in Canada (yes/no), Indigenous status (First Nations, Métis or Inuk (Inuit)/non-Indigenous), marital status (married, living common-law, widowed, divorced, separated, single/never married), household income (less than $20,000, $20,000–$39,000, $40,000–$59,000, $60,000–$79,000, $80,000–$99,000, $100,000 or more), highest level of education attained by respondent (less than high school graduation, high school diploma, postsecondary certificate/diploma, university degree), and highest level of education attained by respondent’s mother and father (less than high school graduation, high school diploma, some postsecondary, postsecondary certificate/diploma, university degree).

### Analysis

Frequency estimates were produced to describe the prevalence of CM and IPV among the study population. Associations between CM and IPV in adulthood were examined using cross-tabulations and logistic regression models that controlled for potential confounders. Two sets of analyses were conducted to address objectives 1 and 2; one based on binary (yes/no) CM variables, and the other to examine severity. The small proportion of respondents with missing data for the CM and/or IPV variables (Table 2) were excluded from the analyses.

**Table 2:** Percentage experiencing intimate partner violence (IPV) in adulthood and childhood maltreatment (CM), by sex, household population aged 18 or older, currently living with a spouse/partner or who had contact with an ex-spouse/partner during last 5 years, Canada, 2014

	Men			Women		
	Sample size	Weighted percent	95% CI	Sample size	Weighted percent	95% CI
			**IPV in adulthood**			
**Any IPV**						
Yes	1684	16.1	(15.1, 17.1)	1911	14.0*	(13.2, 14.8)
No	8743	83.9	(82.9, 84.9)	9389	86.0	(85.2, 86.8)
Missing	181			158		
**Emotional IPV**						
Yes	1563	14.9	(13.9, 15.8)	1808	13.1*	(12.3, 13.9)
No	8896	85.1	(84.2, 86.1)	9521	86.9	(86.1, 87.7)
Missing	149			129		
**Physical IPV**						
Yes	435	4.3	(3.7, 4.9)	572	3.5*	(3.1, 3.9)
No	10,030	95.7	(95.1, 96.3)	10,755	96.5	(96.1, 96.9)
Missing	143			131		
**Sexual IPV**						
Yes	14	F		81	0.5*	(0.3, 0.6)
No	10,456	99.9	(99.8, 100)	11,250	99.5	(99.4, 99.7)
Missing	138			127		
**Physical or sexual IPV**						
Yes	437	4.3	(3.7, 4.9)	582	3.6*	(3.2, 4.0)
No	10,020	95.7	(95.1, 96.3)	10,741	96.4	(96.0, 96.8)
Missing	151			135		
**Emotional IPV and/or physical/sexual IPV**						
Both emotional IPV and physical/sexual IPV	316	3.2	(2.6, 3.7)	479	2.8	(2.4, 3.2)
Emotional IPV, no physical/sexual IPV	1229	11.6	(10.8, 12.5)	1321	10.3*	(9.5, 11.0)
Physical/sexual IPV, no emotional IPV	120	1.2	(0.9, 1.5)	102	0.8	(0.6, 1.0)
No emotional IPV and no physical/sexual IPV	8743	84.0	(83.0, 85.0)	9389	86.1	(85.3, 86.9)
Missing	200			167		
			**Childhood maltreatment**			
**Any CM**^a^						
Yes	2889	26.9	(25.8, 28.0)	3047	25.1*	(24.0, 26.2)
No	7218	73.1	(72.0, 74.2)	8120	74.9	(73.8, 76.0)
Missing	501			291		
**CPA**						
Yes	2513	23.4	(22.3, 24.5)	1771	14.6*	(13.7, 15.4)
No	7612	76.6	(75.5, 77.7)	9431	85.4	(84.6, 86.3)
Missing	483			256		
**CPA severity and frequency**						
Severe and frequent CPA	313	2.7	(2.3, 3.1)	260	2.0*	(1.6, 2.3)
Severe CPA (≤10 times)	783	7.4	(6.8, 8.1)	508	4.1*	(3.6, 4.6)
CPA (excluding severe CPA)	1392	13.1	(12.3, 14.0)	991	8.4*	(7.7, 9.1)
No CPA	7612	76.7	(75.7, 77.8)	9431	85.5	(84.7, 86.4)
Missing	508			268		
**CSA**						
Yes	557	4.7	(4.2, 5.2)	1705	13.5*	(12.7, 14.3)
No	9823	95.3	(94.8, 95.8)	9550	86.5	(85.7, 87.3)
Missing	228			203		
**CSA severity and frequency**						
Severe and frequent (≥ 3 times) CSA	93	0.7	(0.5, 0.9)	406	3.2*	(2.7, 3.6)
Severe CSA (≤2 times)	180	1.6	(1.3, 1.9)	507	4.1*	(3.6, 4.5)
Sexual touching (excluding severe CSA)	282	2.5	(2.1, 2.9)	786	6.2*	(5.7, 6.8)
No CSA	9823	95.3	(94.8, 95.8)	9550	86.5	(85.7, 87.3)
Missing	230			209		
**Childhood exposure to IPV**						
Yes	624	5.4	(4.9, 6.0)	888	7.0*	(6.3, 7.7)
No	9729	94.6	(94.0, 95.1)	10,377	93.0	(92.3, 93.7)
Missing	255			193		
**Frequency of childhood exposure to IPV**						
More than 10 times	276	2.2	(1.9, 2.6)	478	3.5*	(3.0, 4.0)
Three to 10 times	348	3.2	(2.8, 3.6)	410	3.5	(3.0, 4.0)
Never, once or twice	9729	94.6	(94.0, 95.1)	10,377	93.0	(92.3, 93.7)
Missing	255			193		
**Co-occurrence of CM**						
No CM	7218	73.6	(72.4, 74.7)	8120	75.4	(74.3, 76.4)
CPA only (no CSA/Childhood exposure to IPV)	1780	17.0	(16.1, 18.0)	791	6.9*	(6.3, 7.6)
CSA only (no CPA/Childhood exposure to IPV)	209	2.0	(1.6, 2.3)	889	7.3*	(6.6, 7.9)
Childhood exposure to IPV only (no CPA/CSA)	137	1.3	(1.0, 1.6)	259	2.4*	(1.9, 2.8)
Two or 3 types of CM	700	6.1	(5.6, 6.7)	1032	8.1*	(7.4, 8.7)
Missing	564			367		

Source: Statistics Canada: 2014 General Social Survey: Victimization

CPA: childhood physical abuse; CSA: childhood sexual abuse

^a^ CPA, CSA or childhood exposure to IPV

* Significantly different from men (p < 0.05)

F too unreliable to be published

All analyses were based on weighted data. Weights created by Statistics Canada ensured that the data were representative of the Canadian population in 2014. To account for the survey design effect of the GSS, standard errors, coefficients of variation, and 95% confidence intervals were estimated using the bootstrap technique [38]. Differences between estimates were tested for statistical significance, which was established at the *p* < 0.05 level. All analyses were conducted using SAS 9.4 (SAS Institute Inc.; Cary, North Carolina, USA).

Preliminary analyses considered CM in relation to the three types of IPV in adulthood: physical, sexual, and emotional. Sample size was not sufficient to examine associations with sexual IPV separately; therefore, sexual and physical IPV were combined. Owing to the overlap between physical/sexual and emotional IPV, we examined associations between CM and three outcomes: emotional IPV alone (no physical/sexual IPV); physical/sexual IPV alone (no emotional IPV); and both emotional IPV and physical/sexual IPV. Sample size was insufficient to determine associations between CM and physical/sexual IPV with no emotional IPV. When the outcomes were emotional IPV with no physical/sexual IPV or physical/sexual IPV and emotional IPV, associations with CM were similar. Therefore, only the results for any IPV (physical, sexual, or emotional) are presented.

To examine sex differences in associations between CM and IPV, logistic regression was used to test for interactions with sex and CM. A significant interaction indicates that the strength of the association between CM and IPV differs by sex (for example, if a statistically significant interaction greater than 1.0 is found between CM and IPV for being female, the association with IPV is stronger for women than for men). When significant interactions terms were found, we reported on relative risks based on rates since it is not appropriate to compare odds ratios across models.

## Results

### Estimates of IPV

Among people aged 18 years or older currently living with a spouse/partner or who had contact with an ex-spouse/partner in the last 5 years, a slightly higher percentage of men than women reported any IPV victimization (physical, sexual or emotional): 16.1% versus 14.0% (Table 2).

Men were more likely than women to report physical and emotional IPV, while women were more likely to report sexual IPV. Based on Statistics Canada guidelines, the sample size for men who reported sexual IPV was too small for the estimate to be published; therefore, the combined percentage reporting physical/sexual IPV is presented.

The percentages of men and women reporting both emotional IPV and physical/sexual IPV were similar (3.2 and 2.8%, respectively). Reporting physical/sexual IPV with no emotional IPV was rare for both sexes (1.2 and 0.8%, respectively). Among the population who reported physical/sexual IPV, it was very common to also report emotional IPV; 72.7% among men and 77.4% among women (data not shown).

### Estimates of CM

Men were slightly more likely than women to report any CM: 26.9% versus 25.1%. A higher percentage of men than women reported CPA (23.4% versus 14.6%), but women were more likely to report CSA (13.5% versus 4.7%) and childhood exposure to IPV (7.0% versus 5.4%). Similarly, men were more likely than women to report severe/frequent CPA, while women were more likely to report severe/frequent CSA and frequent childhood exposure to IPV. A higher percentage of women than men reported co-occurrence of two or more forms of CM (8.1% versus 6.1%).

### Associations between CM and IPV

For both sexes, those who had experienced any CM were more likely to report being victimized by IPV in adulthood (Table 3). Among men reporting any CM, 23.4% reported IPV in adulthood, compared with 13.2% of those who did not experience CM; among women, the corresponding figures were 23.0 and 10.9%. Men and women who had experienced each type of CM (CPA, CSA and childhood exposure to IPV) had higher rates of IPV than did those who had not.

**Table 3:** Prevalence of and adjusted odds ratios for experiencing any intimate partner violence (IPV) in adulthood, by sex and childhood maltreatment (CM), household population aged 18 or older, currently living with a spouse/partner or who had contact with an ex-spouse/partner during last 5 years, Canada, 2014

	Any IPV in adulthood				
	%	95% CI	Odds		95% CI
	**Men**				
**Any CM**^a^					
Yes	23.4*	(21.2, 25.6)	1.8 *		(1.5, 2.1)
No (reference)	13.2	(12.1, 14.3)	1.0		…
**CPA**					
Yes	23.3 *	(21.0, 25.6)	1.7 *		(1.4, 2.0)
No (reference)	13.8	(12.7, 14.9)	1.0		…
**CSA**					
Yes	25.1 *	(20.3, 29.9)	1.8 *		(1.4, 2.5)
No (reference)	15.5	(14.5, 16.6)	1.0		…
**Childhood exposure to IPV**					
Yes	27.7 *	(23.0, 32.4)	2.0 *		(1.5, 2.6)
No (reference)	15.3	(14.3, 16.3)	1.0		…
**Simultaneously controlling for other CM types**					
**CPA** (reference no CPA)			1.5 *		(1.3, 1.8)
**CSA** (reference no CSA)			1.5 *		(1.1, 2.1)
**Childhood exposure to IPV** (reference no childhood exposure to IPV)			1.7 *		(1.3, 2.2)
	**Women**				
**Any CM**^a^					
Yes	23.0 *	(21.1, 25.0)	2.1 *		(1.8, 2.5)
No (reference)	10.9	(10.0, 11.8)	1.0		…
**CPA**					
Yes	25.9 *	(23.0, 28.7)	2.2 *		(1.8, 2.7)
No (reference)	11.8	(11.0, 12.7)	1.0		…
**CSA**					
Yes	24.8 *	(22.1, 27.4)	2.0 *		(1.7, 2.5)
No (reference)	12.2	(11.3, 13.1)	1.0		…
**Childhood exposure to IPV**					
Yes	26.6 *	(22.6, 30.7)	2.1 *		(1.6, 2.8)
No (reference)	13.0	(12.2, 13.8)	1.0		…
**Simultaneously controlling for other CM types**					
**CPA** (reference no CPA)			1.8 *		(1.4, 2.2)
**CSA** (reference no CSA)			1.6 *		(1.3, 1.9)
**Childhood exposure to IPV** (reference no childhood exposure to IPV)			1.5 *		(1.2, 2.0)

Source: Statistics Canada: 2014 General Social Survey: Victimization

CPA: childhood physical abuse; CSA: childhood sexual abuse

^a^ CPA, CSA or childhood exposure to IPV

* Significantly different from reference (*p* < 0.05)

Note: Odds are adjusted by age group, Canadian-born, Indigenous status, marital status, household income, highest level of education of respondent, and highest level of education of respondent’s father and mother

… not applicable

The associations between the three individual types of CM and IPV in adulthood persisted when potential socio-demographic confounders were taken into account. For both sexes, the adjusted odds ratios for reporting IPV were significant for each type of CM. When we simultaneously controlled for the co-occurrence of CM, all three types remained statistically significant for both sexes, demonstrating that each type is independently associated with IPV in adulthood.

### Associations between severity of CM and IPV

When severity/frequency of CM was considered, a dose-response relationship between CPA and IPV in adulthood emerged for both sexes—as the frequency/severity of CPA increased, so did the likelihood of reporting IPV (Table 4). The highest IPV rates were among those who had experienced severe/frequent CPA (31.3% for men and 31.8% for women), substantially exceeding the rates among those who had not experienced any CPA (13.8% for men and 11.8% for women).

**Table 4:** Prevalence of and adjusted odds ratios for experiencing any intimate partner violence (IPV) in adulthood, by sex and co-occurrence of childhood maltreatment (CM) and CM severity, household population aged 18 or older, currently living with a spouse/partner or who had contact with an ex-spouse/partner during last 5 years, Canada, 2014

	Any IPV in adulthood			
	%	95% CI	Odds	95% CI
	**Men**			
**CPA severity and frequency**				
Severe and frequent CPA (> 10 times)	31.3 *^a^	(24.1, 38.4)	2.3 *	(1.6, 3.3)
Severe CPA (≤10 times) (reference 3)	25.2 *	(21.0, 29.5)	1.7 *	(1.4, 2.2)
CPA (excluding severe CPA) (reference 2)	20.2 *	(17.2, 23.2)	1.6 *	(1.2, 1.9)
No CPA (reference 1)	13.8	(12.7, 14.9)	1.0	…
**CSA severity and frequency**				
Severe and frequent (≥3 times) CSA	28.8 *	(16.8, 40.8)	1.9	(0.9, 3.9)
Severe CSA (≤2 times) (reference 3)	23.8 *	(15.0, 32.6)	1.6	(0.9, 2.8)
Sexual touching (excluding severe CSA) (reference 2)	24.7 *	(18.1, 31.2)	1.9 *	(1.3, 2.8)
No CSA (reference 1)	15.5	(14.5, 16.6)	1.0	…
**Frequency of childhood exposure to IPV**				
More than 10 times	24.7 *	(17.8, 31.6)	1.6 *	(1.0, 2.5)
Three to 10 times (reference 2)	29.8 *	(23.2, 36.4)	2.3 *	(1.7, 3.2)
Never, once or twice (reference 1)	15.3	(14.3, 16.3)	1.0	…
**Co-occurrence of CM**				
No CM (reference 1)	13.2	(12.1, 14.3)	1.0	…
CPA only (no CSA/Childhood exposure to IPV - reference 2)	22.3 *	(19.5, 25.1)	1.7 *	(1.4, 2.1)
CSA only (no CPA/Childhood exposure to IPV - reference 3)	23.1 *	(15.2, 30.9)	2.0 *	(1.2, 3.3)
Childhood exposure to IPV (no CPA/CSA - reference 4)	29.3 *	(18.5, 40.0)	2.4 *	(1.4, 4.1)
Two or 3 types of childhood maltreatment	25.8 *	(21.5, 30.1)	2.2 *	(1.7, 2.9)
	**Women**			
**CPA severity and frequency**				
Severe and frequent CPA (> 10 times)	31.8 *^a^	(23.8, 39.7)	2.7 *	(1.7, 4.2)
Severe CPA (≤10 times) (reference 3)	30.4 *^a^	(24.7, 36.0)	2.6 *	(1.9, 3.7)
CPA (excluding severe CPA) (reference 2)	22.3 *	(18.8, 25.7)	2.0 *	(1.6, 2.5)
No CPA (reference 1)	11.8	(11.0, 12.7)	1.0	…
**CSA severity and frequency**				
Severe and frequent (≥3 times) CSA	28.3 *^a^	(22.4, 34.1)	2.3 *	(1.7, 3.3)
Severe CSA (≤2 times) (reference 3)	29.3 *^a^	(23.9, 34.6)	2.5 *	(1.8, 3.5)
Sexual touching (excluding severe CSA) (reference 2)	20.0 *	(16.7, 23.3)	1.6 *	(1.2, 2.1)
No CSA (reference 1)	12.2	(11.3, 13.1)	1.0	…
**Frequency of childhood exposure to IPV**				
More than 10 times	30.7 *^a^	(24.9, 36.5)	2.5 *	(1.8, 3.5)
Three to 10 times (reference 2)	22.7 *	(17.2, 28.1)	1.8 *	(1.3, 2.6)
Never, once or twice (reference 1)	13.0	(12.2, 13.8)	1.0	…
**Co-occurrence of CM**				
No CM (reference 1)	10.9	(10.0, 11.8)	1.0	…
CPA only (no CSA/Childhood exposure to IPV - reference 2)	19.8 *	(16.1, 23.5)	1.8 *	(1.3, 2.3)
CSA only (no CPA/Childhood exposure to IPV - reference 3)	17.5 *	(14.5, 20.5)	1.5 *	(1.1, 2.0)
Childhood exposure to IPV (no CPA/CSA - reference 4)	21.2 *	(14.9, 27.6)	2.1 *	(1.4, 3.3)
Two or 3 types of childhood maltreatment	31.1 *abc	(27.0, 35.2)	3.1 *	(2.4, 4.0)

Source: Statistics Canada: 2014 General Social Survey: Victimization

CPA: childhood physical abuse; CSA: childhood sexual abuse

* Significantly different from reference 1 (*p* < 0.05)

^a^ Significantly different from reference 2 (*p* < 0.05)

^b^ Significantly different from reference 3 (*p* < 0.05)

^c^ Significantly different from reference 4 (*p* < 0.05)

Note: Odds are adjusted by age group, Canadian-born, Indigenous status, marital status, household income, highest level of education of respondent, and highest level of education of respondent’s father and mother

… not applicable

Among women, a dose-response relationship emerged between severity/frequency of CSA and IPV. By contrast, among men, there were no statistically significant differences by severity/frequency of CSA.

Also, for women, a dose-response relationship was observed between the frequency of childhood exposure to IPV and IPV in adulthood; this was not the case for men.

Women who had experienced two or more types of CM had a higher likelihood of reporting IPV in adulthood than did those who had experienced only one of the three types. However, among men, having experienced multiple types of CM was not associated with a higher rate of IPV; rates were similar among those who had experienced two or more types of CM and those who had experienced one type.

The bivariate associations between the severity/frequency CM variables and being victimized by IPV in adulthood persisted when controlling for potential socio-demographic confounders.

### Sex differences in associations between CM and IPV

To examine sex differences, we combined the sexes, and using logistic regression, we tested for interactions between sex and CM in relation to reporting IPV in adulthood, while controlling for potential socio-demographic confounders. A significant sex interaction was observed for CPA—the association between CPA and IPV in adulthood was stronger for women than for men. Women who reported CPA were 2.2 times more likely to experience IPV in adulthood than were those who did not report CPA (25.9/11.8) (Table 3). For men, the disparity was smaller, with those reporting CPA being 1.7 times more likely to experience IPV in adulthood than were those who did not report CPA (23.3/13.8).

Significant sex interactions were also observed for CPA severity. Compared with men who reported no CPA, experiencing IPV in adulthood was 2.3 times higher (31.3/13.8) for those reporting severe and frequent CPA, 1.8 times higher (25.2/13.8) for severe CPA, and 1.5 times higher (20.2/13.8) for CPA, excluding severe CPA (Table 4). Associations were stronger for women—compared with those reporting no CPA, experiencing IPV in adulthood was 2.7 higher for severe and frequent CPA, 2.6 for severe CPA, and 1.9 for CPA, excluding severe CPA.

Similarly, men who reported at least two types of CM were 2.0 times more likely to experience IPV in adulthood than those who reported no CM. For women, the gradient was steeper, with those reporting at least two types of CM being 2.9 times more likely to experience IPV in adulthood.

## Discussion

This study, based on a large, representative sample of the Canadian population in 2014, found that CM is associated with IPV in adulthood. For both sexes, CPA, CSA and childhood exposure to IPV were independently associated with subsequent IPV. Associations between CPA and IPV were stronger for women than for men. Among women, a dose-response relationship emerged between each of the three types of CM and IPV in adulthood—increases in the severity and frequency of the CM were associated with increases in the likelihood of reporting IPV; among men, a dose-response relationship was observed only for CPA. For women, but not men, having experienced two or more types of CM was more strongly associated with IPV than was experiencing one type.

Consistent with results of a study based on data from a 2012 Canadian survey [43], the present analysis found that women were more likely than men to report CSA and childhood exposure to IPV, while men were more likely than women to report CPA.

The present study found that men were slightly more likely than women to report IPV in adulthood. Other studies have also found that women are equally, or somewhat less likely, than men to report IPV, particularly when IPV is broadly defined to include any type of hitting and/or emotional IPV [48, 49]. However, it is important to note that women are at increased likelihood of experiencing severe/frequent IPV, as well as serious consequences, such as psychological harm, physical injuries and mortality [50–52].

Many other studies have found that IPV in adulthood is more common among people who have experienced CSA [5–18] and CPA [5–7, 9, 10, 12–22]. Fewer studies have examined IPV in relation to other types of CM, but associations have been found for childhood exposure to IPV [5, 13, 16–20, 23–25], neglect [5, 6, 14, 15, 20, 22], and childhood emotional abuse [5, 6, 14, 15, 20]. Nonetheless, results have been inconsistent, with some studies finding a null association between specific types of CM and IPV [5–7, 12–14, 19, 20, 22, 24, 26–29]. This likely reflects differences in the definitions of CM and IPV, the reference periods for CM and IPV, the control variables in the analyses (including simultaneously controlling for multiple types of CM), the study population, and the sample size.

CM severity in relation to IPV in adulthood has been studied less frequently. It has been suggested that exposure to CM, regardless of severity, increases the risk of subsequent abuse [33]. Two meta-analyses of associations between CM and IPV published in 2019 (one based solely on men and one on both sexes) [30, 31] reported weak but statistically significant associations. Failure to account for severity of the CM may have masked the full nature of the association. According to our analysis, severity was an important factor, especially for women.

Much of the research examining associations between CM and IPV has been based solely on samples of women; sex differences have rarely been examined. It has been suggested that the lack of studies on male IPV victimization reflects the more serious consequences experienced by females victimized by IPV [30]. Similar to our results for women, other studies revealed a dose-response relationship between the number of types of CM and IPV in adulthood [5, 9, 12, 15, 18–20, 28, 32]. We found that associations between CPA and IPV were stronger among women, but the association was also significant among men. Additional work is needed using mixed gender samples in which multiple types of CM and IPV are considered. Most studies based on men have focused solely on IPV perpetration [30]. However, studies including males victimized by IPV are important to inform both the general population and health care professionals of the importance of recognizing that men also experience victimization and for the development of interventions aimed at reducing male IPV victimization [30].

The most common framework for interpreting IPV risk factors is the ecological model [3, 4]. This model extends beyond individual characteristics to encompass family, community, and societal factors. It recognizes that individuals evolve within a set of nested environmental structures and attempts to identify mechanisms that contribute to vulnerability to repeated abuse. Exo-system and macro-system variables, such as a scarcity of resources, lack of social support, and cultural tendencies to blame the victim, may increase the likelihood that people who experienced CM will be exposed to IPV in adulthood, independent of their individual characteristics. For example, lack of access to affordable housing and well-paying jobs may increase the likelihood that individuals with histories of CM will enter and maintain intimate relationships with people who are violent toward them. CM is associated with numerous negative long-term economic outcomes including unemployment, low income, homelessness, and lack of job skills [44]. This is further exacerbated by experiences of adult IPV victimization which have been shown to inhibit job stability [53].

Another ecological consideration is that the abusive and violent behaviours towards children and partners may not be condemned universally. If people grow up and continue to live in a social milieu where violence is sometimes regarded as acceptable, exposure to frequent and severe violence in childhood may be a marker for exposure to violence in adulthood. This is consistent with the social learning theory model which contends that CM may serve as a model for future interpersonal relationships [54]. Children who experience abuse or have a caregiver who is abused may perceive violence as a normal part of a relationship. Initiatives such as the #MeToo movement (https://metoomvmt.org/about/) may have a role in decreasing IPV. The movement may reduce the likelihood of accepting abuse of any kind in intimate relationships, regardless of past experiences of violence. Research based on Canadian administrative data found an increase in reporting sexual assaults to the police after the emergence of #MeToo, particularly cases involving a known perpetrator [55].

The ecological model also incorporates the idea that the family environment in which the CM initially occurred may be associated with future abuse. A systematic review of risk and protective factors for revictimization after CSA found that the perception of parental care acted as a buffer, reducing the risk of revictimization [56].

A 2019 study [57] found that positive childhood experiences reduced the risk of poor mental health in adulthood, independent of childhood abuse and neglect and household dysfunction (such as parental substance abuse). The measures of positive childhood experiences included items such as participation in community traditions, a sense of belonging in high school, and social support from friends. These findings demonstrate the utility of the ecological model when investigating associations between CM and other negative outcomes in adulthood such as IPV.

### Strengths and limitations

This study has notable strengths: it is based on a large, representative sample of the Canadian population; multiple behaviour-specific items were used to measure CM and IPV; and these measures have been shown to have greater validity and reliability than broad, subjectively defined items [41, 42, 58–60]. As a result, it was possible to examine CM severity in relation to IPV in adulthood and to test for sex differences in associations.

However, the analysis has limitations that should be considered when interpreting the findings:

- All information was based on retrospective self-reports. Recall bias may have influenced the observed associations between CM and IPV. People who experienced violence in adulthood may more readily recall CM, which could have inflated associations. On the other hand, those who experienced CM may be less likely to report IPV in adulthood because of habituation to violence in general; this would weaken associations.
- Individuals who experienced CM and/or IPV may have been reluctant to disclose these experiences in a survey.
- The items used to measure IPV in adulthood were asked only of respondents with a current spouse or common-law partner and/or those who had contact with an ex-spouse/common-law partner in the past 5 years. IPV that occurred in dating relationships was excluded. Evidence suggests that the association between CM and IPV is stronger for dating couples than for married ones [31].
- The GSS questionnaire did not include measures of childhood emotional abuse and neglect, which have been shown to be associated with IPV [5, 6, 14, 15, 20, 22].
- In the regression models, it was not possible to control for some potentially important confounders (such as overall family dysfunction) due to unavailability in the GSS. It has been hypothesized that family dysfunction may be more strongly related to negative outcomes than is CM, but some research indicates that each is independently related to dysfunction in adulthood [61]. Socioeconomic status during childhood was partially controlled by inclusion of highest level of education of the father and mother in the regression analyses; family income data would have offered a more complete control for childhood socioeconomic status.
- The coverage of the GSS excludes people who are homeless and those living in institutions—populations for which experiences of CM and IPV are more prevalent.
- The extent to which the low GSS response rate (52.9% for the provinces; 58.7% for the territories) affects associations between CM and IPV is unknown. An analysis of data from Statistics Canada’s Canadian Community Health Survey (an ongoing annual health survey) reported steady declines in response rates over time. The characteristics of respondents and non-respondents differed in ways that cannot be fully corrected via weighting [62]. Furthermore, vulnerable populations (for instance, lower socioeconomic status and poor health) [62, 63] are the least likely to respond to surveys.
- Previous studies have found that CM is associated with IPV perpetration, particularly among men [8, 30, 32, 64, 65], and reciprocal IPV [8, 32, 65]. However, the GSS did not include questions on IPV perpetration so it was not possible to examine CM in relation to IPV perpetration.
- It is possible that there are subpopulations for which the associations between CM and IPV in adulthood are stronger such as Indigenous peoples [23]. The child’s relationship to the perpetrator may also be an important factor [5]; children who experience abuse from a parent or caregiver may be at a heighted risk of experiencing IPV compared with those whose relationship to the abuser was more distant. We did not have adequate sample to examine interactions with these variables.

Future research should examine other types of CM such as neglect and emotional maltreatment in relation to IPV in adulthood. A survey with a larger sample size would be instrumental in identifying subpopulations with elevated associations between CM and IPV who are particularly in need of early interventions. More research on the effectiveness of community-based interventions such as fostering a sense of belonging to school and community traditions is warranted [57].

## Conclusion

Numerous studies have found that both CM and IPV are associated with an increased risk of a wide range of physical and mental health conditions [66–68]. The negative outcomes of physical IPV are immediate and apparent, including injury, physical disability, and in severe cases, mortality [66]. However, emotional IPV also has serious ramifications; emotional IPV is strongly related to numerous adverse conditions, including chronic disease and poor mental health [69]. The association between CM and IPV in adulthood merits attention because of cumulative effects—lifespan studies have shown that individuals who experience numerous incidents of abuse exhibit the highest levels of impairment [70, 71]. Costs to the health care system and the burden to individuals experiencing multiple incidents of abuse underscore the importance of intervention programs to eradicate CM and IPV. Interventions aimed at promoting healthy relationships and providing emotional support and coping mechanisms to children and families in abusive situations are key components to ending the cycle of violence and preventing IPV in adulthood [72].

## Data Availability

The data are available for analysis from Statistics Canada. MS conducted the analyses as an employee of Statistics Canada, under a contact between the Public Health Agency of Canada and Statistics Canada.

## Abbreviations

CI: confidence interval
CPA: childhood physical abuse
CSA: childhood sexual abuse
GSS: General Social Survey
IPV: intimate partner violence
